# Supplementary material for: Measured greenhouse gas budgets challenge emission savings from palm-oil biodiesel
Source: Nat Commun. 2020 Feb 27;11:1089. doi: 10.1038/s41467-020-14852-6 (PMC7046764; doi:10.1038/s41467-020-14852-6)
Supplement: Supplementary file 1 — Supplementary Infornation [file 41467_2020_14852_MOESM1_ESM.pdf]

Supplementary Information for

MEASURED GREENHOUSE GAS BUDGETS CHALLENGE EMISSION SAVINGS FROM PALM-OIL BIODIESEL

by Meijide *et al*.

Supplementary Figures

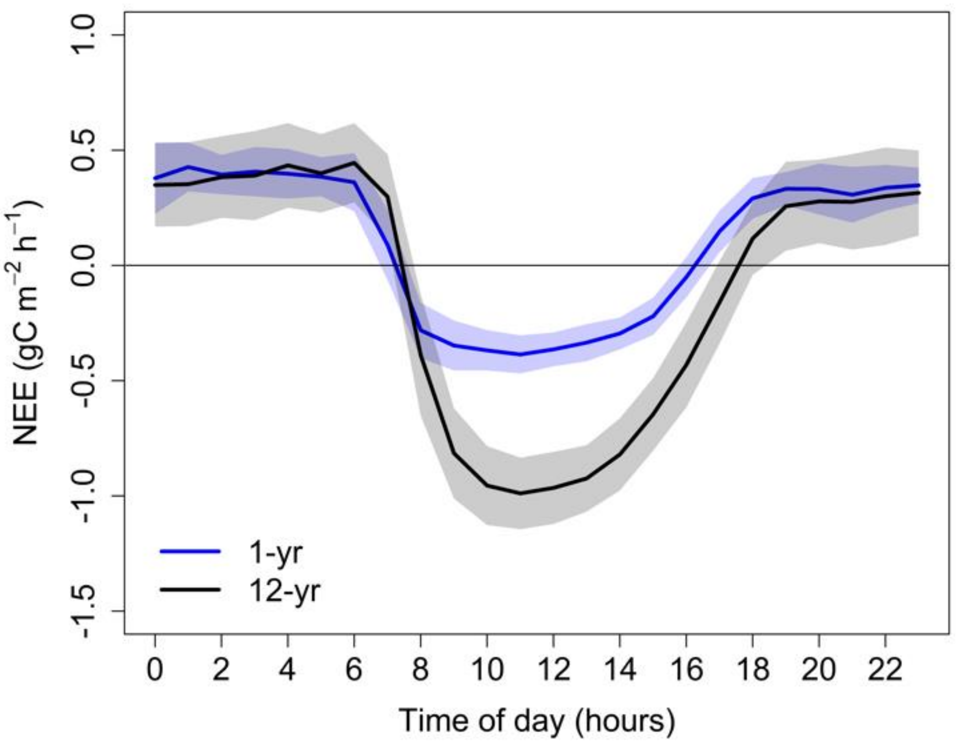

Supplementary Figure 1: Mean diurnal cycles of net ecosystem exchange. Mean diurnal cycles of net ecosystem exchange (NEE) for the 1-year old (1-yr; blue) and 12-year old (12-yr; black) oil palm plantations. Shaded areas indicate standard deviations. Negative and positive fluxes indicate CO_2 uptake and emission, respectively.

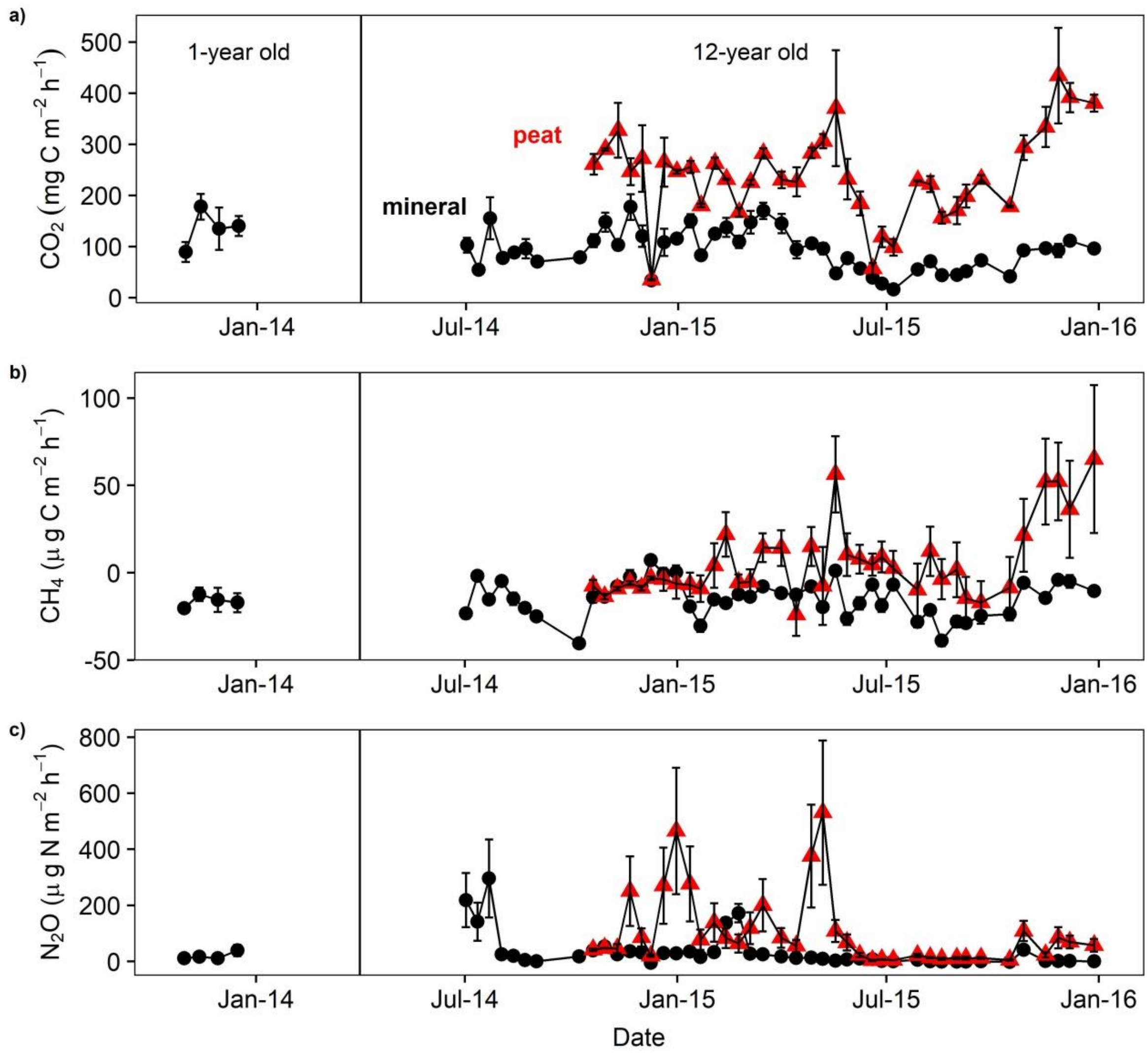

Supplementary Figure 2: Soil greenhouse gas fluxes from plantations. Soil greenhouse gas fluxes from young and mature (1- and 12-year old) oil palm plantations on mineral and peat soils. Mean soil respiration (a; CO_2) and methane (b; CH_4) and nitrous oxide (c; N_2O) fluxes in the 1-year old (left side) and 12-year old (right side) plantations. Data in the 12-year old plantation include measurements on mineral (black dots) and adjacent peat soils (red triangles). Bars represent standard errors, with n = 3 for the 1- year old plantation, n = 4 for mineral soils in the 12-year old plantation and n = 3 for peat soils in the 12-year old plantation.

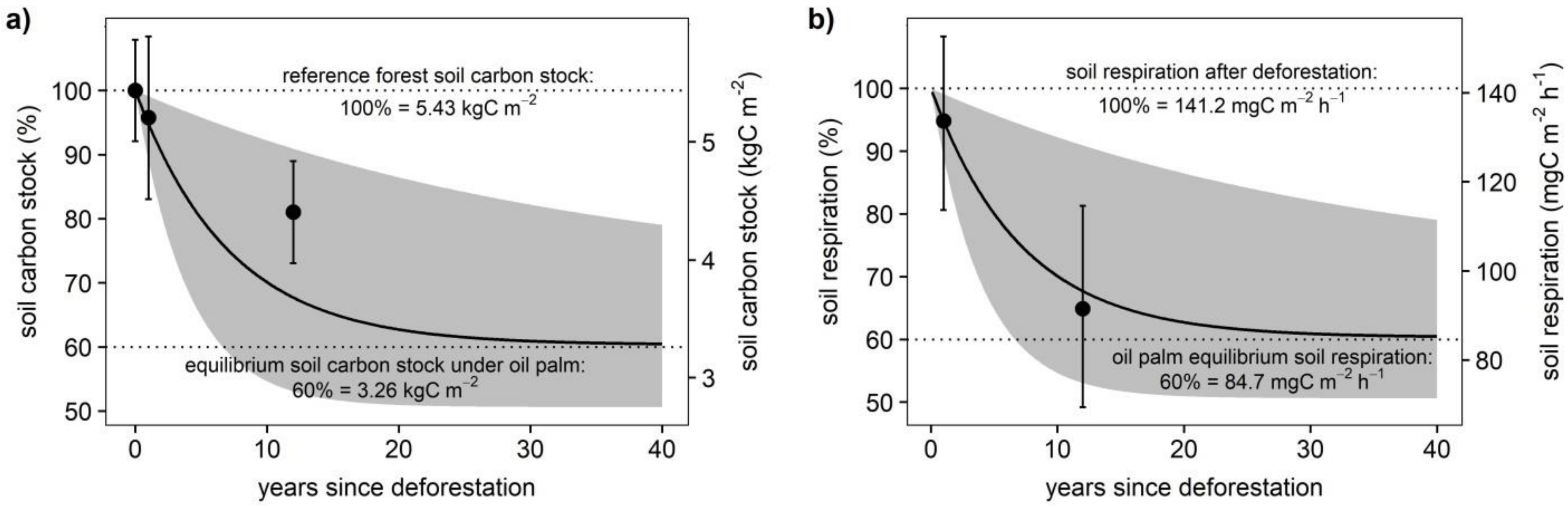

Supplementary Figure 3: Carbon stocks and soil respiration following deforestation. Evolution of soil carbon stocks (a), expressed as a percentage of the original soil carbon stock in reference forest (left axis) and as absolute values (right axis). Measurements in reference forest in the study region were performed by Guillaume et al.,[1] and were used to calibrate the soil organic decay function by Van Straaten et al.,[2].This decay function was used to calculate the equilibrium soil carbon stock under oil palm plantations, which is reached approx. 30-35 years after deforestation. Evolution of soil respiration (b), expressed as a percentage of the soil respiration right after deforestation (left axis) and as absolute values (right axis). The measurements in the 1-year old plantation were used to fit the data to the decay function by Van Straaten et al.,[2]. This decay function was used to calculate equilibrium soil respiration. In both figures, lines correspond to decay functions, black dots to measured values and error bars to standard errors.

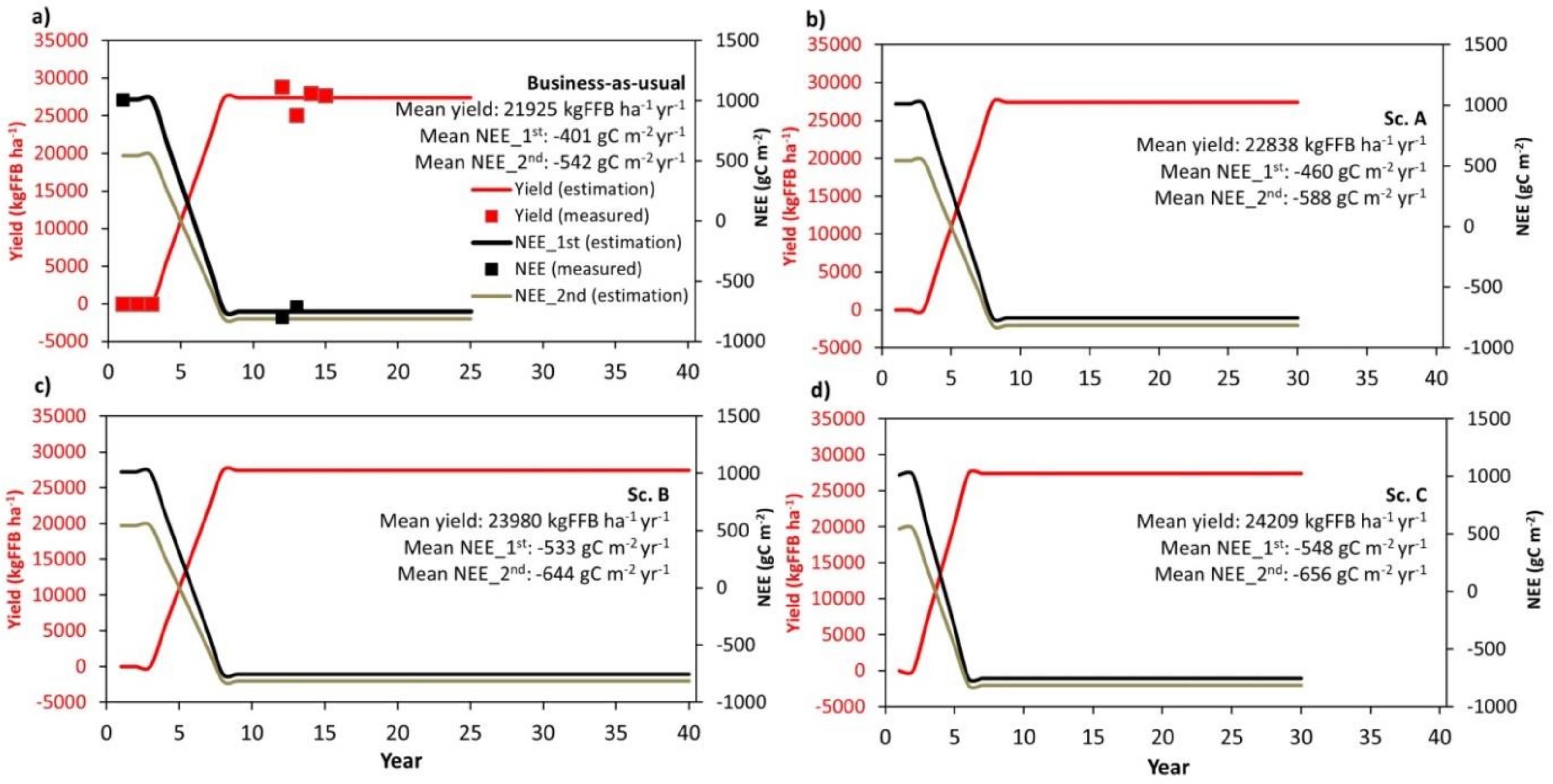

Supplementary Figure 4: Yield and net ecosystem exchange in the rotation cycles. Trends in yield (red) and net ecosystem exchange (NEE, black) over the life cycle of oil palm plantations under different management scenarios. All scenarios assume oil palm cultivation on mineral soils. Business-as-usual assumes a plantation life cycle of 25 years (panel a), while the alternative management scenarios assume extensions of the life cycle to 30 years (panel b, scenario A), 40 years (panel c, scenario B) and to 30 years with the use of earlier-yielding oil palm varieties (panel d, scenario C). Scenarios for NEE are presented for both first and second rotation cycle plantations. Negative and positive NEE values indicate CO_2 uptake and emission, respectively. Data points are measured values whereas lines indicate life cycle estimations. Yield was assumed to be equal in first and second rotation cycle plantations.

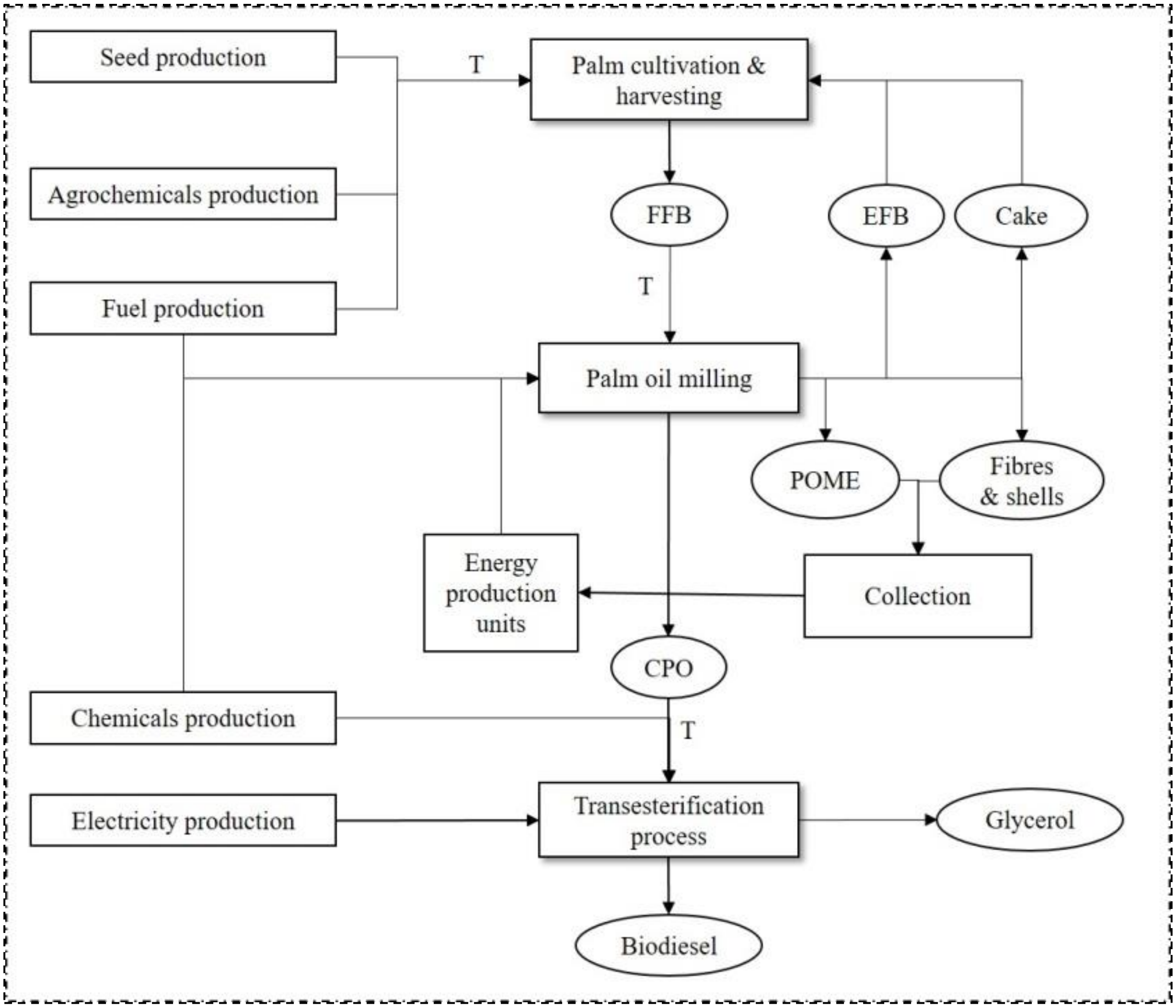

Supplementary Figure 5: System boundaries for palm-oil biodiesel production. The system boundaries of our analysis are represented in this simplified scheme by the outer dashed box. Circles represent products while boxes represent processes. Land-use change related emissions are excluded in the scheme. FFB - fresh fruit bunches, EFB - empty fruit bunches, CPO - crude palm oil, and T - transport. Cake is the residue left after oil is extracted from the kernel.

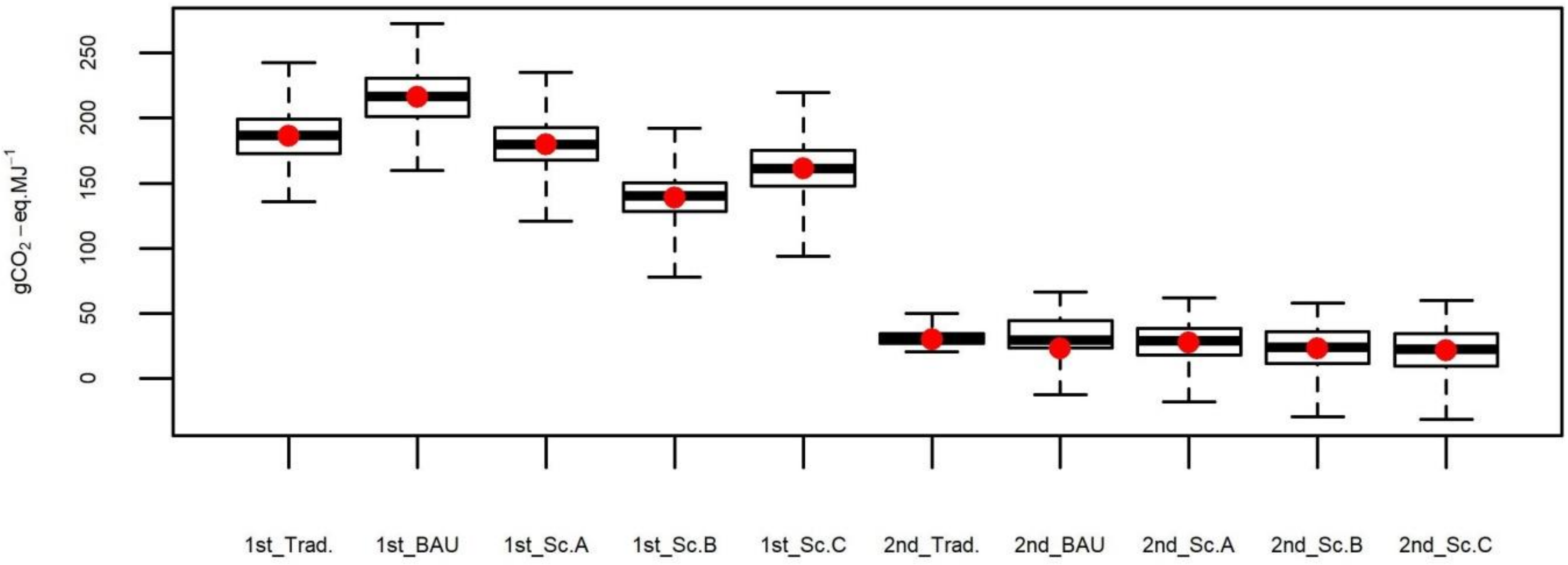

Supplementary Figure 6: Uncertainty analysis for the different scenarios. Uncertainties in the greenhouse gas emissions (in gCO_2-eq. MJ^{-1}) for the different scenarios Emission ranges in g CO_2-equivalents per Megajoule (gCO_2-eq.MJ^{-1}) for the first (1st_) and second (2nd_) rotation cycles for the traditional Life Cycle Analysis (Trad.), business-as-usual (BAU) and the three additional scenarios: scenario A (Sc.A; 30-yr cultivation cycle), scenario B (Sc. B; 40-yr cultivation cycle), and scenario C (Sc. C; 30-yr cultivation cycle with earlier-yielding varieties). These uncertainties include those derived from the different processes, i.e. cultivation, oil milling, processing, use and land-use change related emissions, estimated from Monte Carlo analysis. Boxplots indicate the lower quartile, median and upper quartile, with whiskers extending to the minimum and maximum values. Red dots indicate the estimated mean values.

Supplementary Tables

Supplementary Table 1: Ecosystem carbon fluxes for young and mature plantations. Net ecosystem productivity (NEP) is estimated as net ecosystem exchange (NEE) plus yield; yield in the young plantation was zero. NEP is expressed for both carbon (NEP_C) and carbon dioxide (NEP_{CO2}). Positive values indicate a carbon source from the ecosystem to the atmosphere and negative values a carbon uptake. Errors in NEE and NEP represent 5% of measured fluxes as explained in the Methods section.

	C fluxes from (positive values) and to (negative value) the ecosystem			
	**NEE**	**Yield**	**NEP_C**	**NEP_{CO2}**
	(g C m^{-2} yr^{-1})			(g CO_2-eq. m^{-2} yr^{-1})
Young plantation (1-year old)				
8 months	636 ± 32	---	---	---
1 year	1012 ± 51	---	1012 ± 51	3711 ± 186
Mature plantation (12-year old)				
Year 1	-799 ± 40	851	52 ± 40	192 ± 146
Year 2	-709 ± 35	949	240 ± 35	880 ± 130

Supplementary Table 2: Soil and ecosystem greenhouse gas fluxes. Mean and annual (± SE) soil nitrous oxide (N_2O) and methane (CH_4) fluxes, and carbon dioxide (CO_2) fluxes from the soil (soil respiration; SR) are provided in CO_2 equivalents (CO_2-eq.) Net ecosystem productivity (NEP_{CO2}) and the contribution to the net global warming potential (GWP_{net}) are provided at the ecosystem (Eco) and soil (Soil) levels, for the 1- and 12- year old oil palm plantation as well as for mineral and peat soils in the 12- year old oil palm plantation. For the 1-year old plantation, the less frequent temporal measurements did not allow for estimating annual fluxes. Therefore, we used the soil N_2O emissions measured in smallholder plantations in the study area[21]. Soil CH_4 fluxes from the 1-year old plantation were assumed to be the same as in the mature plantation. For the peat soils of the 12- year old oil palm plantation, we did not measure CO_2 fluxes at the ecosystem level, and therefore fluxes were estimated as NEP_{CO2} = Net ecosystem exchange (NEE)mineral – SRmineral + SR peat + Yield. Different letters in mean fluxes indicate significant differences at $P < 0.01$ according to the Mann-Whitney U test.

	N_2O			CH_4			CO_2				GWP_{net}	
							SR			NEP_{CO2}	Eco	Soil
	mean	annual		mean	annual		mean	anual				
	N	N	GWP_{N2O}	C	C	GWP_{CH4}	C	C	GWP_{CO2}			
	(µgN_2O-N m^{-2} h^{-1})	(gN_2O-N m^{-2} yr^{-1})	(gCO_2-eq. m^{-2} yr^{-1})	(µgC m^{-2} h^{-1})	(gC m^{-2} yr^{-1})	(gCO_2-eq. m^{-2} yr^{-1})	(mgC m^{-2} h^{-1})	(gC m^{-2} yr^{-1})	(gCO_2-eq. m^{-2} yr^{-1})	(gCO_2-eq. m^{-2} yr^{-1})	(gCO_2-eq. m^{-2} yr^{-1})	(gCO_2-eq. $m^{-2}$$yr^{-1}$)
1-yr old; Mineral (n = 4)	19.7 ± 7.9[a]	0.11 ± 0.05[I]	51.5 ± 23.4	-17.0 ± 2.2[a]	-0.13 ± 0.02	-4.3± 0.7	133.9 ± 19.0[a]	---	---	3711 ± 186[IV]	3758 ± 187	---
12-yr old; Mineral (n = 45)[2]	31.1 ± 21.2[a]	0.33 ± 0.17[II]	154.5 ± 79.6	-14.7 ± 4.6[a]	-0.13 ± 0.02	-4.3 ± 0.7	91.7 ± 22.1[a]	885 ± 173	3245 ± 634	536 ± 344[IV]	686 ± 353	3395 ± 639
12-yr old; Peat (n = 37)[3]	104.4 ± 93.6[b]	0.95 ± 0.87[III]	444.9 ± 407.4	6.2 ± 24.0[b]	0.00 ± 0.18	0.0 ± 6.0	239.8 ± 46.1[b]	1969 ± 227	7220 ± 832	4511 ± 1071	4596 ± 1146	7665 ± 927

[I]Data from Hassler et al., 2017[3].
[II]Annual fluxes calculated from measurements from 2-07-2014 until 27-06-2015.
[III]Annual fluxes calculated from measurements from 20-10-2014 until 28-10-2015.
[IV]Value ± uncertainty estimated via bootstrapping approaches as explained in Meijide et al., 2017[4].

Supplementary Table 3: Detailed greenhouse gas emissions for all scenarios. Direct and indirect greenhouse gas emissions (gCO_2-eq. MJ^{-1}) over the life cycle of palm-oil biodiesel from first-rotation cycle and second-rotation cycle oil palm plantations. Traditional life cycle analysis (LCA), which does not include any biogenic carbon dioxide (CO_2) emissions occurring along the life cycle (i.e. the sinks during cultivation and the emissions produced from biological products: fibers and shells, palm oil milling effluent - POME - and biodiesel combustion). Business-as-Usual includes the ecosystem CO_2 and methane fluxes (combined as CO_2-eq. under "ecosystem emissions"), as well as nitrous oxide (N_2O) measured in the field during the cultivation phase and the biogenic CO_2 released along the different phases of the life cycle. 1st and 2nd rotation cycles differ in ecosystems emissions and regarding the inclusion of land-use change related emissions, which are only considered in the 1st rotation cycle; all further emissions are considered to be identical. Traditional LCA and Business-as-usual consider a rotation cycle of 25 years. Scenario A considers an expansion of the oil palm life cycle to 30 years. Scenario B considers an expansion of the oil palm to 40 years. Scenario C takes into account oil palm life cycle to 30 years and early yielding palm varieties. The table is divided in four stages: oil palm cultivation, palm oil milling, biodiesel production, and biodiesel use. No biogenic emissions from palm-oil product use are considered in the traditional LCA. Negative values represent CO_2 sinks.

**Phase**	**Inputs**	**Traditional LCA**	**Business as Usual**	**Business as Usual - allocation by revenue**	**Scenario A (30 years)**	**Scenario B (40 years)**	**Scenario C (30 years + early yielding)**
				gCO_2-eq.MJ^{-1}			
Cultivation	N-Fertilizers	9.6	9.6	9.1	9.4	9.2	9.1
	Phosphate-Fertilizers	1.1	1.1	1.0	1.0	1.0	1.0
	Potassium-Fertilizers	0.5	0.5	0.5	0.5	0.5	0.5
	Glyphosate	0.2	0.2	0.2	0.2	0.2	0.2
	Labours	2.9	2.9	2.8	2.8	2.7	2.6
	Transport	0.2	0.2	0.2	0.2	0.2	0.2
	N_2O direct emissions	6.9	6.9	6.5	6.8	6.6	6.7
	Ecosystem emissions 1st cycle	---	-76.8	-72.6	-84.5	-93.3	-95.0
	Ecosystem emissions 2nd cycle	---	-103.6	---	-107.9	-112.7	-113.6
	**Subtotal 1st cycle**	**21.4**	**-55.3**	**-52.3**	**-63.5**	**-72.9**	**-74.6**

	**Subtotal 2nd cycle**	**21.4**	**-82.4**	**---**	**-86.9**	**-92.3**	**-93.2**
Oil Milling	Hexane and other chemicals	0.1	0.1	0.1	0.1	0.1	0.1
	Fibers and shell	---	29.7	28.8	29.7	29.7	29.7
	Diesel	0.5	0.5	0.5	0.5	0.5	0.5
	POME	---	1.8	1.8	1.8	1.8	1.8
	Transport	0.0	0.0	0.0	0.0	0.0	0.0
	BoP	0.7	0.7	0.0	0.7	0.7	0.7
	**Subtotal**	**1.2**	**32.7**	**31.2**	**32.7**	**32.7**	**32.7**
Production	Methanol and other chemicals	2.5	2.5	2.2	2.5	2.5	2.5
	Electricity	4.0	4.0	3.5	4.0	4.0	4.0
	Transport	0.6	0.6	0.6	0.6	0.6	0.6
	BoP	0.1	0.1	0.1	0.1	0.1	0.1
	**Subtotal**	**7.2**	**7.2**	**6.3**	**7.2**	**7.2**	**7.2**
Use	Biodiesel	---	74.7	74.7	74.7	74.7	74.7
	**Subtotal**	**---**	**74.7**	**74.7**	**74.7**	**74.7**	**74.7**
Land-use change related emissions	Land-use change	132.6	132.6	125.4	106.1	75.8	100.1
	Foregone sequestration	23.7	23.7	22.4	22.7	21.6	21.4
	**Subtotal**	156.3	156.3	176.1	128.8	97.4	121.5
TOTAL	Cultivation	21.4	21.4	20.3	21.0	20.4	20.3
	Sinks 1st cycle	---	-76.8	-72.6	-84.5	-93.3	-95.0
	Sinks 2nd cycle	---	-103.8	---	-107.9	-112.7	-113.6
	Oil Milling	1.2	32.7	31.2	32.7	32.7	32.7
	Processing	7.2	7.2	6.3	7.2	7.2	7.2
	Use	---	74.7	74.7	74.7	74.7	74.7
	Land-use	156.3	156.3	147.8	128.8	97.4	121.5
	**Net emissions 1st cycle**	**186.2**	**215.6**	**207.6**	**179.9**	**139.1**	**161.5**
	**Net emissions 2nd cycle**	**29.9**	**32.5**	**---**	**27.6**	**22.6**	**21.3**
**GHG savings (%) 1st cycle**		**-98**	**-129**	**-121**	**-91**	**-48**	**-72**
**GHG savings (%) 2nd cycle**		**68**	**65**	**---**	**71**	**76**	**77**

Supplementary Table 4: Main cultivation parameters considered in the scenarios. Description of the main parameters considered during the cultivation phase in the business-as-usual scenario and the alternative management scenarios to minimize greenhouse gas emissions: A (30-yr cultivation cycle), B (40-yr cultivation cycle), and C (30-yr cultivation cycle with early yielding varieties), both for first- and second-rotation cycle oil palm plantations. For yield, fertilizers and herbicide applications the presented values are the values for the indicated years along the rotation cycle, as well as the yearly means for the whole life cycle. Management similar to smallholder plantations during the non-productive periods and similar to intensively-managed plantations during the productive periods are assumed.

Scenario	**1st year production**	**Plateau year**	**Life time** (years)	**Year**	**Yield** (kg ha^{-1} yr^{-1})	**N-fertilizer** (kgN ha^{-1} yr^{-1})	**K-fertilizer** (kgK ha^{-1} yr^{-1})	**P-fertilizer** (kgP ha^{-1} yr^{-1})	**Glyphosate** (ml ha^{-1} yr^{-1})
Business-as-usual	4	8	25	1-3	0	88	73	38	0
				4	5841	110	102	40	450
				5	10962	132	132	43	900
				6	16444	153	161	45	1350
				7	21925	174	191	48	1800
				8-25	27406	196	220	50	2250
				**mean**	**21925**	**174.4**	**190.6**	**47.6**	**1800**
Scenario A	4	8	30	1-3	0	88	73	38	0
				4	5841	110	102	40	450
				5	10962	132	132	43	900
				6	16444	153	161	45	1350
				7	21925	174	191	48	1800
				8-30	27406	196	220	50	2250
				**mean**	**22838**	**178.0**	**195.5**	**48.0**	**1875**
Scenario B	4	8	40	1-3	0	88	73	38	0
				4	5841	110	102	40	450
				5	10962	132	132	43	900
				6	16444	153	161	45	1350
				7	21925	174	191	48	1800
				8-40	27406	196	220	50	2250
				**mean**	**23980**	**182.5**	**201.6**	**48.5**	**1969**
Scenario C	3	6	30	1-2	0	88	73	38	0
				3	6852	115	110	41	563
				4	13703	142	147	44	1125
				5	20555	170	183	47	1688
				6-30	27406	196	220	50	2250
				**mean**	**24209**	**183.4**	**202.9**	**48.6**	**1988**

Supplementary References

1. Guillaume, T. et al. Carbon costs and benefits of Indonesian rainforest conversion to plantations. *Nature Communications* **9**, 2388 (2018).
2. Van Straaten, O. et al. Conversion of lowland tropical forests to tree cash crop plantations loses up to one-half of stored soil organic carbon. *Proceedings of the National Academy of Sciences of the USA* **112**, 9956-9960 (2015).
3. Hassler, E., Corre, M.D., Kurniawan, S. & Veldkamp, E. Soil nitrogen oxide fluxes from lowland forests converted smallholder rubber and oil palm plantations in Sumatra, Indonesia. *Biogeosciences* **14**, 2781-2798 (2017).
4. Meijide, A. et al. Controls of water and energy fluxes in oil palm plantations: Environmental variables and oil palm age. *Agricultural and Forest Meteorology* **239**, 71-85 (2017).
